# Stiff person case misdiagnosed as conversion disorder: A case report

**DOI:** 10.22088/cjim.8.4.329

**Published:** 2017

**Authors:** Saeed Razmeh, Amir Hasan Habibi, Farzad Sina, Elham Alizadeh, Monireh Eslami

**Affiliations:** 1 Department of Neurology, Rasoul Akram Hospital, Iran University of Medical Sciences, Tehran, Iran.

**Keywords:** Stiff person syndrome, Gait disorder, Conversion disorder

## Abstract

**Background:**

Stiff person syndrome (SPS) is a rare neurological disease resulting in stiffness and spasm of muscles. It initially affects the axial muscles and then spread to limb muscles. Emotional stress exacerbated the symptoms and signs of the disease. The pathophysiology of the disease is caused by the decreased level of the glutamic acid decarboxylase (GAD) activity due to an autoantibody against GAD that decreases the level of gamma-aminobutyric acid (GABA). In this paper, we present a case of atypical presentation of SPS with lower limb stiffness misdiagnosed as conversion disorder.

**Case presentation:**

We report a patient with atypical presentation of SPS with lower limb stiffness and gait disorder misdiagnosed as conversion disorder for a year. Her antithyroid peroxidase antibody (anti-TPO Ab) level was 75 IU (normal value: 0–34 IU). Intravenous immunoglobulin (IVIG) was administered (2gr/kg, 5 days) for the patient that showed significant improvement in the follow-up visit.

**Conclusion:**

It is essential that in any patient with bizarre gait disorder and suspicious to conversion disorder due to the reversibility of symptoms, SPS and other movement disorder should be considered.

**S**tiff person syndrome (SPS) is a rare neurological disease results in stiffness and spasm of muscles. Emotional stress which is usually progressive exacerbated the symptoms and signs of the disease. The SFS initially affects the axial muscles and then spreads to limb muscles (1, 2, 3). The pathophysiology of the disease is caused by decreased levels of GAD activity due to autoantibody against GAD leading to low GABA levels and increased glutamic acid level which in turn increase the rigidity of muscles (4). It is usually idiopathic but in 5% of cases is a paraneoplastic neurological disorder and also associated with autoimmune conditions such as type 1 diabetes mellitus, Hashimoto’s thyroiditis, vitiligo, pernicious anemia, systemic lupus erythematous and celiac disease (5). We report a case of atypical presentation of SPS with lower limb stiffness misdiagnosed of conversion disorder for a year and responded favorably to IVIG. 

## Case presentation

A 52-year-old woman referred to our clinic with the complaint of gait disturbance and rigidity and muscle spasm of lower limbs since last year. Her muscle stiffness began from the distal lower extremity and progressed to proximal and ultimately became so severe that she had difficulty walking and climbing stairs and eventually became wheelchair bound. She said that it seemed stress and fatigue worsened her symptoms.

The patient had experienced various tests in the different clinics including rheumatology, orthopedy, and psychiatry with varied diagnosis and finally diagnosed of conversion and started taking antidepressants. Her medical history was significant for Hashimoto’s thyroiditis and hypertension. Her family history and review of systems were noncontributory. Her medications included sertraline 50mg, levothyroxine 25mcg, atenolol 100 mg once a day and pregabalin 75 twice daily. 

On physical examination, there was no malaise, fever, lymphadenopathy and organomegaly. In the neurological examination of the lower limbs, movement in all direction was limited and rigidity in distal and proximal muscles was detected. Deep tendon reflexes were normal in the upper and lower extremities and plantar response was bilateral flexor. 

In laboratory examinations, including creatine kinase, complete blood count, erythrocyte sedimentation rate, and C- reactive protein and rheumatoid factor level were normal. The thyroid function tests, antithyroglobulin and thyroid-stimulating hormone receptor antibody also were normal. Her anti-TPOAb level was 75 IU (normal value: 0–34 IU). Serum anti-GAD level was 72(normal value < 1) and the patient’s tumor markers and paraneoplastic tests were normal. 

In electromyography (EMG) continuous motor unit discharge and simultaneous co-contraction were observed in lower extremities. Bilateral mammography, abdominopelvic and chest CT for occult cancer was normal. IVIG was administered (100gr, 2gr/kg/5 days) for the patient with significant improvement observed from the admission period and 2 months later in the follow-up visit.

## Discussion

The first description of SPS was by Moersch and Woltman in 1956, a rare condition that affects females more than males. The main clinical presentation includes various muscle involvements in the trunk, limbs and neck. Usually the rigidity and stiffness begin insidiously in axial muscle and over time progress involving proximal muscle, additionally sound impulses, emotional stress and tactile Stimulus exacerbate muscle spasm of the patient. (2, 5). In our case, rigidity and spasticity began in lower distal limbs then spread to proximal and caused recurrent falls that is extremely rare in SPS. Occasionally, paroxysmal autonomic dysfunction, including diaphoresis, papillary dilatation, tachypnea, tachycardia, hypertension, and hyperpyrexia, have been reported in SPS and may result in sudden death. (6) GABA is an inhibitory neurotransmitter in the brain and spinal interneurons decrease in SPS due to inhibition of GABA synthesis by glutamic acid decarboxylase autoantibody that induces continuous motor neuronal discharge (4, 7). 

This autoantibody is present in about 60% of SPS patients. In our case, the serum anti–GAD Ab was positive. SPS has been associated with autoimmune diseases, such as type 1 diabetes mellitus, autoimmune thyroiditis, epilepsy, pernicious anemia, and vitiligo, also 5% of cases is due to paraneoplastic disorder of breast cancer, small cell lung cancer and lymphoma, therefore, full work-up for paraneoplastic and autoimmune disease should be done (8-10). Psychiatric disorder includes depression, anxiety, panic disorder, post-traumatic stress and conversion disorder associated with SPS. 

The exact mechanism of such an association remains unknown, it may be the result of impaired GABAergic inhibition caused by the anti-GAD antibodies. In our case after extensive investigations, depression disorder was diagnosed and gait disorder was misdiagnosed with conversion disorder 1 year ago. Psychogenic gait disorders are common and are the major manifestations in patients with psychogenic movement disorders. But it may be over diagnosed and missed the organic problems. 

There are many types of medical treatment for SPS including oral and intrathecal baclofen, diazepam, corticosteroid and IVIG. Occasionally rituximab (anti-CD-20 monoclonal antibody) attacks B lymphocytes and has been reported to lead the clinical improvement of SPS (11). Our case responds dramatically with IVIG. In conclusion, it is essential that in any patient with bizarre gait disorder and suspicious to conversion disorder, SPS should be considered to prevent delay in diagnosis and long-term neurologic disability.
